# Distribution, abundance and traditional management of *Agave potatorum* in the Tehuacán Valley, Mexico: bases for sustainable use of non-timber forest products

**DOI:** 10.1186/1746-4269-10-63

**Published:** 2014-09-03

**Authors:** América Delgado-Lemus, Alejandro Casas, Oswaldo Téllez

**Affiliations:** 1 Centro de Investigaciones en Ecosistemas, (CIECO), Universidad Nacional Autónoma de México (UNAM), Apartado Postal 27–3, Santa María Guido, C.P. 58090 Morelia, Michoacán, México; 2 UBIPRO, Facultad de Estudios Superiores, Iztacala (UNAM) Avenida de los Barrios, S. N., Tlalnepantla, Estado de México, México

**Keywords:** *Agave potatorum*, Maguey, *Mescal*, Non-timber forest products, Plant management, Sustainable use, Tehuacán valley

## Abstract

**Background:**

*Agave* species have been used for thousands of years in the Tehuacán Valley, but the current mescal production has great impact on populations of the most used species. Harvesting of *A. potatorum* takes place before sexual reproduction and the over-extraction put local populations at high risk. In the community of San Luis Atolotilán (SLA), mescal has been produced for one century but the growing mescal trade is leading to intensified agave extraction. Our study evaluated distribution and abundance of *A. potatorum*, extraction rates, management practices and economic importance for SLA households. The unbalanced relation between availability and extraction rates would be an indicator of risk requiring sustainable management strategies. Our case study aspires contributing to analyze general patterns for sustainable use for this and other forest products highly extracted.

**Methods:**

We used bioclimatic modeling to project a map of potential distribution of the species, and ecological sampling to estimate the total availability of harvestable agaves within the territory of SLA. We used participant observation, surveys and semi-structured interviews with producers and households of SLA to document agave uses, technological and socio-economic aspects of mescal production, and to estimate extraction rates of agaves.

**Results:**

Mescal production, medicine and fodder are the most important uses of *A. potatorum.* Its distribution area is nearly 608 ha where annually occur on average 7,296 harvestable plants, nearly 54 to 87% of them being harvested. Mescal production currently is a non-sustainable activity, requiring great changes in patterns of extraction and management adopting sustainable criteria. Local people started management planning to ensure the future availability of agaves, and the ecological information of this study has been helpful in constructing their decisions. Technical support for improving local experiences for managing populations’ recovering is a priority. Interaction of scholars and local people for solving this problem is already taking place and strengthening this process may be determinant for successful results.

**Conclusions:**

Strategies for protecting particular populations, temporal substitution of agave species for mescal production, implementation of restoration and organization for fear commerce are needed for improving sustainable use of *A. potatorum*.

## Background

The genus *Agave* comprises species with high ecological, economic and cultural value in Mexico. Out of 200 *Agave* species existing in the world 150 are found in Mexico [1], with a wide distribution in a variety of landscapes. The ancient use of agaves by local cultures continues up to the present: nearly half of the species are used for their fibers in traditional manufacturing of clothes, cords, nets, textile knitting, tools, and handcrafts; some others are used as food, in traditional medicine, as construction material, and living fences [2]. Nearly 53 species are useful for preparing alcoholic beverages such as “pulque” (fermented sap agave beer) and mescal, a spirit distilled from baked and fermented agave stems [2-4]. All these agave products commonly offer significant goods for direct consumption and monetary incomes to the livelihoods of numerous Mexican communities, especially in arid and semi-arid areas. But the use of most species continues without regards their future availability or socio-ecological problems associated to their use. These are particularly the cases of those intensely cultivated species like the tequila agave (*Agave tequilana* var. *azul*) whose production involves progressive substitution of large areas of forests by plantations throughout Mexico, as well as extensive propagation of clones with resulting narrow genetic diversity populations [5,6]. But it is also the case of most species of agave, which are extracted from wild populations without management practices focused on recovering their affected populations. Except nine species that are cultivated and twelve that receive some incipient management type, populations of 44 agave species of Mexico are extracted from the wild are threatened because of their non-regulated harvesting (notice that the sum of the agave species referred to in this sentence is higher than 53; this is because a species may be wild, incipiently managed and/or cultivated [4]), a situation that could determine gradual local extinctions of the species if alternative management techniques are not fully developed. We analyze in this study the case of *Agave potatorum*, a semelparous agave species endemic to the states of Oaxaca and Puebla in central Mexico, where the Tehuacán Valley is located. It is the case of one particularly important species that is endangered, but the methods, analyses and reflections derived from this case study may help for developing diagnosis and designing strategies for other agave species of the Tehuacán Valley as well as dozens of agave species extracted from forests in other regions of Mexico. It may also be helpful for analysing the cases of other forest resources highly extracted for commercialization and that are also in high risk [7].

The TehuacánValley lodges an extraordinary biologic and cultural diversity. It is the region with the greatest *Agave*s species richness in Mexico [1], embracing 23 species, with which humans have had a long history of interaction [8,9]. In this region, human cultural history is between 12,000 and 14,000 years old, according to archaeological studies conducted in caves of the area by MacNeish [9]. In addition to the abundant remains of nets and clothes confectioned with fiber of agave leaves from strata of 7,000 years of antiquity [10], archaeological studies found remains of chewed agave fibers in caves floor and coprolites of the most ancient strata associated with human occupation of the region, nearly 10,000 years ago [9,11]. The agave remains found suggest the prehistoric use of these plants as food, prepared by roasting the stems, leaf bases and floral escapes [11] and probably flower buds like currently people do. The sweet cooked agave food called “*mezcalli*” (from the Náhuatl term *metl* meaning agave and *izcalli* meaning oven) was cooked like currently for mescal production, baking the stems in underground ovens using heated stones. Archaeologists reported ovens used for this purpose in the Tehuacán Valley as well as in several archaeological sites throughout Mexico [8,12]. The archaeological studies of the Tehuacán valley allowed a complete chronological reconstruction of the prehistory of the region and have been of great importance to characterize processes that led to origins of agriculture and domestication of plants [13], in which agaves had a central role.

Recently, the harvesting activity causing higher impact on wild agave populations is growing with the mescal industry, which has determined extraction techniques and rates that have already decreased and caused extinction of local *Agave potatorum* populations [14]. Development of the mescal industry has been carried out without comprehensive knowledge about ecological, social and economic implications of mescal production. Therefore, it is of great concern to generate information of such processes that might help us to understand the role of mescal production in household economy, the conservation state of local agave populations, the impact of extraction on ecosystems and the management techniques required for its conservation.

We focused our attention on a case study in San Luis Atolotitlán (SLA), Puebla, located in the Tehuacán-Cuicatlán Valley biosphere reserve. Our study aimed to: (1) characterize the distribution and abundance of *Agave potatorum*, (2) analyze the traditional use, management and extraction rates of *A. potatorum,* the economic importance of mescal production and trading, (3) identify risk factors in the current use patterns and the main challenges for constructing sustainable management strategies, and (4) identify critical actions for sustainable use of this and other agaves of Mexico under similar situations, as well as other non-timber forest products with commercial value.

## Material and methods

### Study area

**Figure 1:**
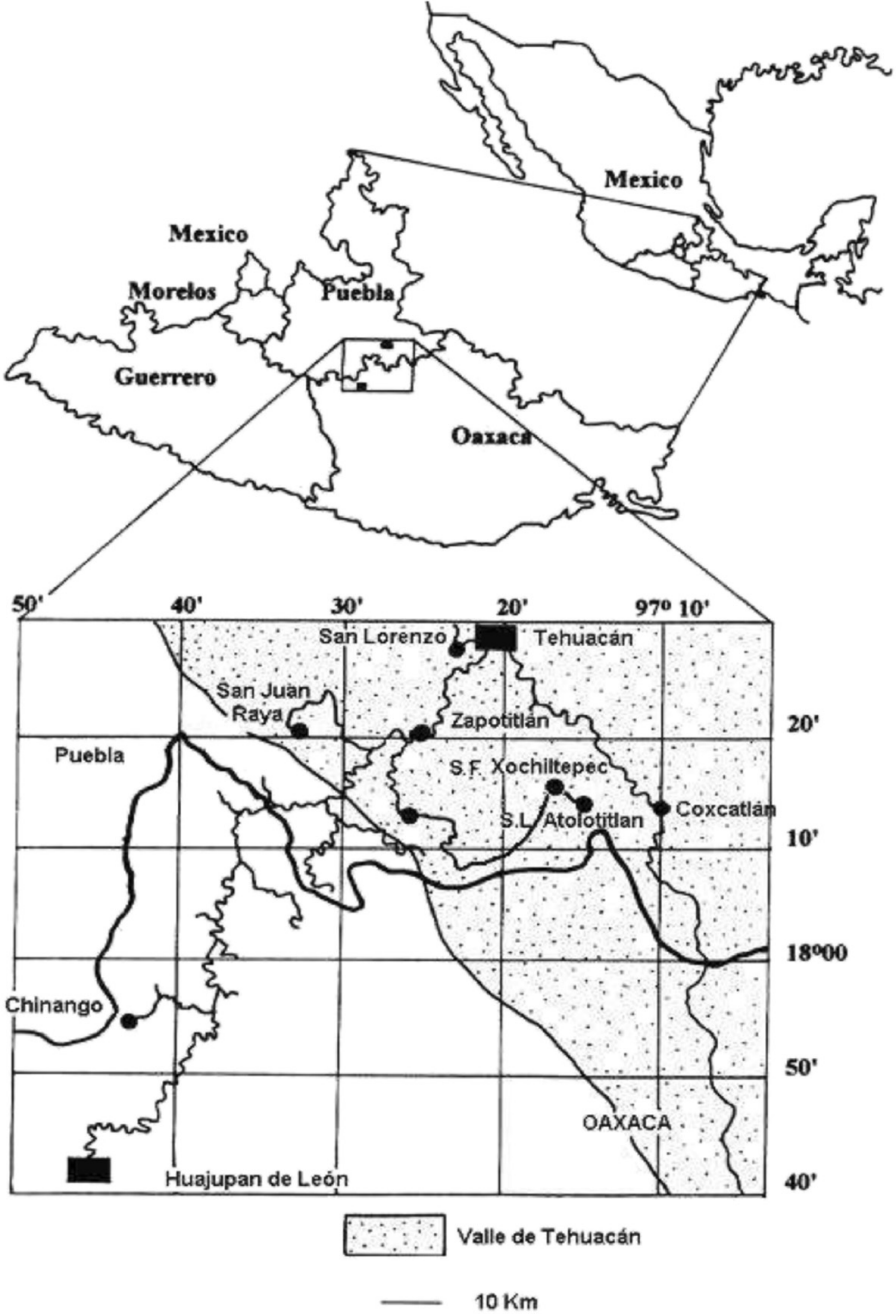
**Study area.** Location of San Luis Atolotitlán and other villages of the Tehuacán-Cuicatlán Valley mentioned in this study.

The Tehuacán-Cuicatlán Biosphere Reserve is located in the southeastern part of the state of Puebla and the northwestern region of the state of Oaxaca, central Mexico (Figure 1). This is the southernmost arid area of Mexico [15] and its aridity is determined by the orographic rain shadow caused by the *Sierra Madre Oriental*. The Biosphere Reserve covers nearly 4,950 km^2^, nearly one half of the whole Tehuacán-Cuicatlán Valley region (10,000 km^2^). The reserve contains a variety of climates, warm climates with annual mean temperature of 21°C and rainfall of 700 to 800 mm in the southeast, semi-warm climates with annual rainfall of 300 to 500 mm in the central and western areas, and temperate climates with 600 mm of annual rainfall in the northwest [16]. A mosaic of plant communities includes 36 association types, described and classified by [17] according to their physiognomy, dominance, and structure.

Our study centered in the case of the village and territory of San Luis Atolotitlán (SLA) with the purpose of developing a methodological tool that could help for analysing the cases of other species and wider spatial scales. SLA is located at the southeast of the state of Puebla (Figure 1), with average elevation of 1900 m [18] comprising a territory of approximately 118 km^2^. The village of SLA was founded after the Mexican revolution in 1915, in the area of the Hacienda de SLA Tultitlanapa. People of SLA are descendants from Náhuatl communities of the surrounding areas. At the time of this study only three older members of the community referred to that their grandparents spoke Náhuatl language. However, there are still numerous terms derived from Náhuatl commonly used by local people; this is the case of “*papalometl*” (meaning “butterfly agave”), the name of *Agave potatorum*. People are mostly peasants dedicated to seasonal agriculture of corn and beans, complementing their diet with nearly 44 species of wild edible plants [19]. Some households are also dedicated to extensive raising of cattle and goats. Almost all women weave handicrafts with palm (*Brahea dulcis*) leaves. Nearly one third of households participate in activities related to mescal production, and a few households are traders or work in clothes factories in Tehuacán (‘maquilas’ factories) [14].

### Climate

According to [16], the climate type registered in the nearest meteorological station in Caltepec, is semi-arid with annual mean temperature above 18°C, a dry station during the winter (November to January) and a rainy season in summer (June-September) with annual precipitation averaging 655 mm. Nevertheless, local people mentioned to have perceived progressive decreasing of rainfall.

### Vegetation

We firstly described and characterized vegetation types of the territory of SLA based on remote perception images, fieldwork to corroborate the physiognomy of vegetation units and vegetation sampling by 500 m^2^ plots in all the vegetation types identified according to [17] throughout the whole territory [19]. We identified eight plant association types: (1) scrubland of *Euphorbia antisyphilitica*, (2) scrubland of *Dasylirion serratifolium*, (3) scrubland of *Gochnatia-Dasylirion*, (4) forest of *Mitrocereus fulviceps*, (5) oak forest, (6) cacti forest of *Polaskia chichipe*, (7) chaparral or mexical of *Dasylirion serratifolium*, (8) scrubland (izotal) of *Beaucarnea purpusii*. We constructed a map of these vegetation types, identifying those where *Agave potatorum* was present, and complemented the records with the “walk in the woods” method, which allowed identifying areas of presence of the agave studied in vegetation types in which previous studies did not identify it. This method also allowed identifying a larger number of geo-referenced points for constructing a bioclimatic collection of data that were used for projecting the map of potential distribution. In addition, we identified and geo-referenced areas where people knew there were agaves some 15 to 20 years ago and became extinct because their extraction for preparing mescal.

### *Agave potatorum*

**Figure 2:**
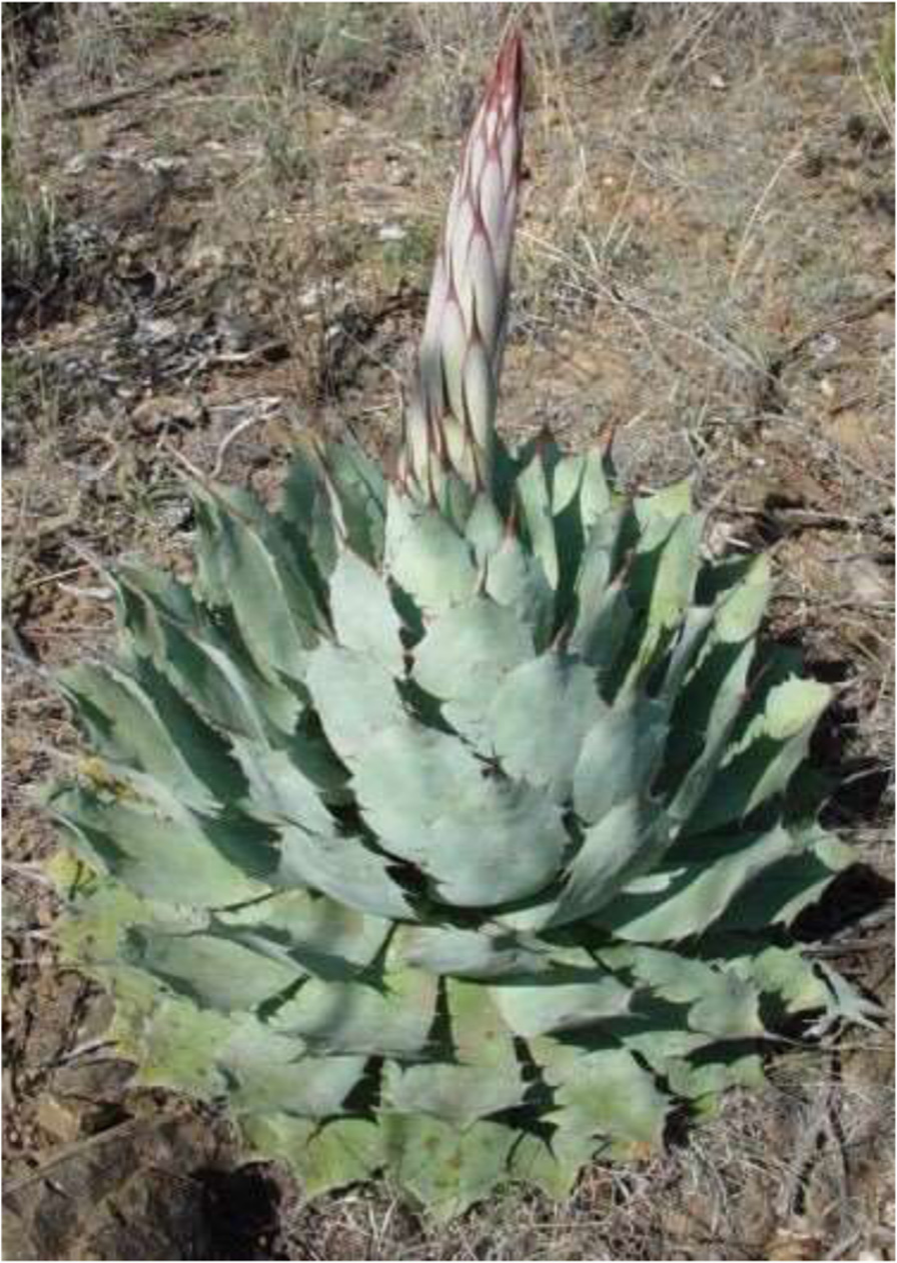
**Aspect of an individual of *Agave potatorum *** **Zucc. showing an early floral escape.**

The individuals of *Agave potatorum* (Figure 2) form relatively small rosettes with 50 to 80 glaucous or light green broad leaves (the name “butterfly agave” makes reference to this feature) with margins undulated to deeply crenate with prominences, mostly 25–40 by 9–18 cm [20]. Their spines are 3 to 4.5 cm long, broad at their base, sinuous, broadly grooved to flat above, with castaneous to grayish brown color. Their inflorescences are 3 to 6 m tall, racemoses with sub-sessile flower clusters or panicles with lateral peduncles [20]. It is a monocarpic species with only sexual reproduction [20]. The species is endemic of semi-arid habitats of the states of Puebla and Oaxaca, growing at elevations from 1240 to 2300 m and its populations have a fragmented distribution.

### Ecological aspects

#### Distribution

In order to estimate the distribution of *A. potatorum* in the territory of SLA, we recorded 40 geographic locations of *A. potatorum* populations in random points of vegetation types where the species was previously identified and those areas that through the method of “walks-in the woods” [21] which allowed identifying and geo-referencing its present and past occurrence. We analyzed data with the bioclimatic model BIOCLIM of the software package ANUCLIM [22], which employs climatic surfaces that are statistically interpolated on the basis of data obtained from a network of meteorological stations. These surfaces include average values of monthly periods, as well as minimum and maximum temperature and precipitation from 6500 stations in Mexico and surrounding areas [23]. A potential distribution map to a high spatial resolution of 0.004 arc seconds (50 × 50 m) was projected using Arcview GIS 3.2 [24], which was also used to calculate the total surface of the potential distribution area. Information from this process was verified in the field.

### Abundance

Abundance of *A. potatorum* was evaluated through 21 sampling plots of 500 m^2^. About 80% of the points were randomly determined within the distribution area documented through vegetation types sampling and complemented by “walks-in the woods” as explained above. Some points recommended by the mescal producers were sampled, since these were identified as preferred harvesting sites.

Plot sites included rosetofilous scrubland of *Dasylirion serratifolium*, scrubland of *Euphorbia antisiphylitica*, scrubland of *Gochnatia hypoleuca* – *Dasylirion serratifolium* and cacti forest of *Mitrocereus fulviceps*. We identified and counted all the mature agaves ready to be harvested (extractable agaves) and multiplied that number by five to calculate availability by hectare. We then averaged data from all plots to estimate a number of extractable agaves/ha and multiplied this figure by the total potential distribution area of *A. potatorum* within the territory of SLA.

### Ethnobotanical study

Through semi-structured interviews [25] to a total of 47 households we obtained the information reported in this study. A total of 30 households (20% of the total in SLA) were selected at random in the village, and with the interviews we documented the traditional uses, amounts and frequency of extraction of *Agave potatorum* plants or other products per year, cultural and economic importance of mescal and other agave products, and information about its management. We complemented this information by participant observation, and informal interviews, which substantially enriched ethnobiological qualitative information on use, preparation, and consumptions forms of agave products, as well as management techniques.

### Extraction

We evaluated the rate of *A. potatorum* extraction through surveys and interviews with mescal production units (a total of 12 of the 15 owners of the production units who directly extract agave plus the 5 persons which in total were identified to be dedicated to extracting and selling agaves to the owners of production units). Interviews focused on documenting the agave extraction techniques, amounts of agaves extracted, frequency of extraction per year, and provenance of agave (from the territory of SLA or from elsewhere), among other aspects. We complemented this information through direct observations and video-recording of the extraction process. Based on this information we estimated the annual extraction rate of *A. potatorum* in SLA.

### Economic importance

We documented economic aspects of mescal production and commercialization through interviews to 12 out of a total of 15 mescal producers identified in SLA at the time of this study. The main topics documented in these interviews included prices and amounts of agave stems for producing mescal, salaries and cost of production of the activity (including fuelwood for the underground ovens, extraction, milling of the cooked agave stems, fermenting process care, and distillation), making explicit the amount and cost of hand labor and other inputs. For investigating aspects of commercialization of the elaborated mescal we interviewed the 12 producers referred to above, as well as nine traders selling mescal in commercial establishments in the village, as well as the 30 local households interviewed who consume this beverage.

## Results

### Ecological aspects

#### Distribution

**Figure 3:**
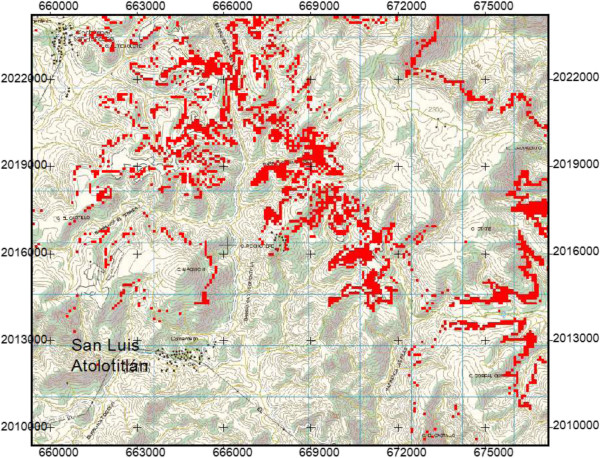
**Map of potential distribution (in red) of *Agave potatorum *** **in the territory of San Luis Atolotitlán, Puebla.**

We found and sampled *Agave potatorum* in eight vegetation types: scrubland of *Euphorbia antisiphylitica*, scrublands of *Dasylirion serratifolium,* scrubland of *Gochnatia- Dasylirion*, columnar cacti forest of *Mitrocereus fulviceps*, columnar cacti forest of *Polaschia chichipe*, izotal of *Beaucarnea purpusii* and *Quercus* forest. Based on this information and records of presence of *A. potatorum* in the field we calculated that the potential distribution of *A. potatorum* covers approximately 608 ha of the territory of SLA (Table 1, Figure 3).

**Table 1:** Average number of individuals of adult plants (extractable for preparing mescal) per vegetation type within the territory of SLA

**Vegetation type**	**Availability individuals/hectare**
Scrubland of *Euphorbia antisyphilitica*	53
Cacti forest of *Mitrocereus fulviceps*	20
Rosetofilous scrubland of *Dasylirion serratifolium*	18
Cacti forest of *Polaschia chichipe*	15
Rosetofilous scrub (Chaparral)	8
Izotal of *Beaucarnea purpusii*	5
*Gochnatia hypoleuca- Dasylirion* scrub	5
Forest relict of *Quercus* sp.	0
AVERAGE	15.38

### Abundance

We recorded the greatest abundance of *A. potatorum* in the scrub of *Euphorbia antisiphylitica* (on average 53 extractable individuals per ha), whereas the least abundance was recorded in oak forests (Table 1). The average density calculated based on sampling conducted was 12 adult plants of *A. potatorum* per ha, which allows estimating a total of 7,296 extractable individuals of *A. potatorum* in the whole territory of SLA at the time of the present study.

### Ethnobotany

#### Uses

*Agave potatorum* is a species with high cultural value for people of SLA. We recorded seven current use categories, as defined by [26], in order of importance: mescal production (the whole stem), food (leaves, stem, flowers and floral scape), medicine (leaves and mescal), fodder (floral scape), construction (floral scape), religious (whole plant and mescal), and ritual (mescal*)* as described in Table 2. Other uses are occasionally practiced, such as the preparation of agave leaves conserves and cooked agave stems and escapes mixed with corn to cook tortillas which are considered emergency food, consumed during scarcity of maize. However, the traditional knowledge of agave uses may be progressively disappearing, as could be identified through the scarce information provided especially by the young women interviewed.

**Table 2:** **Use and management forms of *Agave potatorum *** **recorded in San Luis Atolotitlán**

**Plant part**	**Use**	**Use form**	**Percentage of users**	**Management**
**Stem**	Mescal production	Whole stems are backed inside an earthen pit, grounded, fermented and distilled	100	Extraction of whole individuals from wild populations
**Flower buds**	Food	Flowering buds are boiled or roasted, coocked with eggs or hot chilli sauce	66	People extract the whole scape, to get the flowering buds
**Floral scape**		Roasted over the fire while working in the field	33	Extract the young whole floral scape
**Leaf bases**	Food	People ask for the backed leave bases to the mescal producer and eat them as candy	30	Leave bases remaining from mescal production
	Medicine	An infusion of cooked agave leaf for lung affections	10	
		Applying a piece of fresh leaf (or roasted) directly to wounded area	33	Eventually cutting up one leaf
**Floral escape**	Fodder	Cattle eat the growing scape	40	Cattle eat the early floral scape while it is starting to grow
	Construction	Used in small fences	16	Extract the whole floral scape
**Whole plant**	Religious	Agaves are transplanted from the wild to “little mountains” (*montecitos*) dedicated to the GuadalupeVirgin	10	Each year in December small agave individuals are transplanted from wild populations to home gardens
**Uses of *Mescal***	As medicine	A small glass of mezcal aliviate the stomach-ache, flu symptoms, fever and cold- weat	30	(produced from agave stems)
	Ritual	A small glass of mezcal is offered to the death in the “ofrendas de muertos”	80	

### Management

Mescal producers have to pay nearly $10.00 U.S. dollars per year to the communitarian authorities to have the right of open access to harvest *A. potatorum* plants from wild populations. Less than 10% of people who harvest agaves said to leave the most robust agave individuals to reach sexual maturity, flower and produce seeds. Although producers recognize scarcity of agaves SLA is facing, they cannot stop producing mescal and that is the principal reason why in the last decade they started mixing *A. potatorum* stems with those of other agave species, such as *Agave marmorata* (locally known as *pitzometl*) to fill the oven (*la hornada)*. Recently, they have even started buying *Agave angustifolia* and *A. tequilana* in plantations near the City of Tehuacán.

Since nearly five years ago, people of SLA started actions to propagate *A. potatorum*. They are sowing seeds in a greenhouse and transplanting plants three years old into natural agave populations in local forests. They started restoring degraded populations of *A. potatorum* and implemented actions to reintroduce plants into areas where populations of this species disappeared some years ago. They also protected large reforested areas using nets to safeguard the young plants from cattle.

### Extraction rates

Based on interviews, we calculated an annual extraction rate of 12,331 agaves per year. On average, 356 of them are consumed by households as food: i) 66 floral escapes are cut to be used as food, ii) flower buds from additional 132 agaves are extracted during the flowering season from September to January, iii) nearly 80 agave stems are consumed as fodder by cattle, iv) 32 escapes are cut down for construction, v) six are used to make tools, vi) 40 whole plants are extracted for religious purposes. In addition, on average 158 agave leaves are annually harvested to be used as medicine, but this extraction does not affect survival of individual plants; and vii) the over-extraction of *A. potatorum* in SLA is caused mainly by mescal production. Annual mescal production in SLA needs nearly 11,975 agave plants.

Mescal production is an important economic activity during the dry season, consequently, people extract from wild populations of the community territory nearly 70% of agave plants at reproductive stage. At the time of the study, working with 12 households producing mescal we counted 4,250 plants extracted from the territory of San Luis, but according to the interviews this number may be up to 6,400 plants. The remaining agaves used in mescal production are bought to neighboring villages, mainly San Francisco Xochiltepec and Caltepec. Agave collectors harvest almost all reproductive plants from two sites, “Machiche” which is relatively close to the village and relatively more disturbed than “La Cumbre”, the other site which is more distant to the village.

### Economic importance

**Figure 4:**
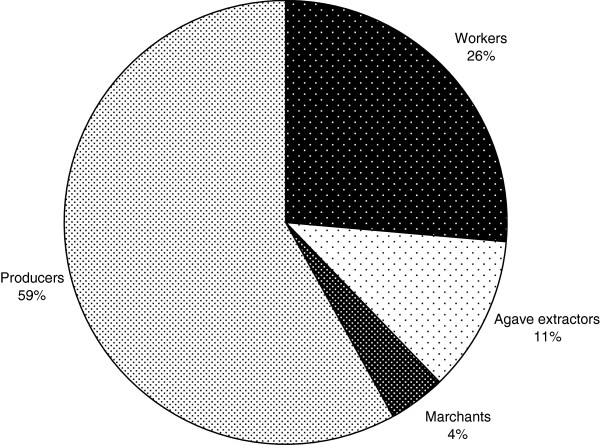
Percentage of earnings corresponding to each mescal production chain in SLA.

Based on the interviews, we estimated that mescal production in SLA during 2007 was approximately 4,000 l (ranging from 3,500 to 4,350 l). In order to produce one liter of *mescal*, on average two adult individuals of *Agave potatorum* and 16.2 kg of firewood are needed. Quality of mescal differs among the 12 local distilleries (locally called *palenques*) studied, depending on the site from which agaves are collected, the tree species used as firewood, and particular procedures and materials used during production.Nearly one third of the households in SLA participate in the process of mescal production, which means that in 60 families at least one member takes part in the process. Based on information from surveys, we estimated annual earnings of the entire village production as $3,921.57 U.S. dollars (the exchange rate at the time of the research being 10.89 Mexican pesos per dollar). Nearly 58% of total income was earned by the producers (Figure 4), who may earn from $64.27 to $422.40 dollars per batch of mescal produced, depending on the number of agaves used and size of the production unit. For instance, 200 agaves can yield from 60 to 180 liters of mescal, depending on the size of the plants and the conditions in which the agave grew (climate, soil, orientation of slope exposure, etc.). Amount of earnings also depends on the cost of certain inputs, including agave stems, workers salary, and the number of batches produced per year (the average being 3.6 batches per household). There are significant differences between the producer with the largest production (he produces mescal at least six times throughout the year), for whom mescal production represents his main income, and other producers for whom mescal production represents one of many activities complementing a multiple use subsistence strategy. Workers receive only minimal income, between $18.36 and $82.64 dollars annually ($41.32 average); they may also be paid with mescal.

## Discussion

*Agave potatorum* represents an important cultural and economic species in SLA. This appreciation is consistent with previous studies on non-timber forest products of the Tehuacán Valley in general and SLA in particular [7,13,14,19]. Such cultural importance is reflected in the multiple uses of *A. potatorum*, in the traditional ecological knowledge about the agave, its biological attributes, morphological and quality variation, including size, sugar content, and yield recognized by local people among sites, as well as ecological interactions with other organisms, as recognized in this ethnobiological study and that conducted by Torres *et al*. [3]. Despite its importance, we documented a significant loss of knowledge related to some *Agave potatorum* uses and forms of preparation, more evident among young women. Young men have good traditional knowledge on this agave species, probably because they do more work in the forest areas and interact directly with *A. potatorum* during mescal production process. The study case of SLA is representative of other communities producing mescal in the region, as these are the cases of Coatepec, Caltepec, and San Francisco Xochiltepec, in the state of Puebla, as well as Santa María Ixcatlán, Santa Cruz Corunda, and Tepelmeme in the state of Oaxaca [3,7], among others. A regional approach to evaluate the problem of availability, extraction rates and traditional management of *A. potatorum* would require methods similar to the one reported in this study. And this method would be similarly helpful for agaves extracted from the wild in other regions, as well as other non-timber forest products under pressure because of their commercialization.

### Extraction rates

*Agave potatorum* is one of the non-timber forest products used with the greatest intensity in SLA an n the whole Tehuacán Valley [3,4,7], mainly because it represents one of the relatively few natural local resources allowing people to obtain monetary incomes [3,4,7]. Mescal producers of SLA harvest or use harvesting of other people totalizing annually nearly 70% of all wild reproductive plants available within the territory of the village. But the impact on some preferred sites could be even higher, since in those areas people use to collect all the reproductive agaves. This over-harvesting is having drastic negative effects on local ecosystems from where agaves are harvested, and also in agave availability, as revealed by the sites where people have identified local extinction. In addition to extraction to satisfy the mescal production, people buy nearly half of agave plants required in neighboring villages, thus affecting populations within other territories of the region. Mescal producers of SLA buy agaves to San Francisco Xochiltepec. Similarly to other related studies [27-29], ours suggests that more attention should be directed towards assessing the effects of agave harvest on the whole plant communities. Harvest of agaves for mescal production affect local wild populations and may determine high risk of disappearing them, but also, other plants and animals interacting with agaves are affected by this activity. In addition, preparing mescal involves the utilization of fuel wood which although regulated in SLA contributes to local deforestation [3,4,30].

### Management

The growing commercial trade of natural products worldwide has resulted in the harvest of increasing volumes from wild plant populations and has therefore generated concern about overexploitation. In the Tehuacán Valley, nearly 180 species of native plant resources have been identified interchanged within and among villages, as well as in the main regional markets [7]. In the case of agaves, 12 species used in mescal production and commercialization are extracted from the wild [14,27,31,32]. Therefore, the information and conclusions of this study may be helpful for attending needs of those agaves.

At the beginning of our study in SLA we identified no management practice other than simple extraction of parts or complete *Agave potatorum* individuals from wild populations. Management based on local ecological knowledge, such as monitoring of specific resources and ecologically focused practices that responded to and managed disturbance and build resilience [33] seemed to be absent. Even though the extraction of *Agave potatorum* focuses on reproductive individuals, based on similar case studies, it is possible to suggest that agave harvest may affect the physiology and vital rates of individuals, change demographic and genetic patterns of populations, and alter community and ecosystem level processes [3,4,27-29]. Extraction may affect not only the capacity of agave populations recovery, of the scrubs surrounding the extractable agaves as reported by Jiménez-Valdéz *et al.*[27] for *Agave marmorata*, but also the ecological interactions with other organisms in the plant communities such as bats, birds and bees feeding on agave flowers, as reported by [34], as well as ants and birds feeding on their seeds [31]. Agaves are also nurse plants for other agaves and succulent plants in their process of recruitment, which is crucial for recovering of the whole biotic community [17,31,35]. Detailed studies of population dynamics [3,4] demonstrated that the extraction rates of *A. potatorum* and its population dynamics are closely related, and have suggested that at least 30% of adult agaves plants occurring in a population should be let standing until the release of seeds, in order to ensure the recovering of the population. These studies have also simulated the optimum forms of propagating seeds and transplanting young plants to particular nurse plants in forest areas in order to increase the success of actions.

By the end of our study we identified local perceptions of risks the agave populations are facing. Local people recognize sites where *A. potatorum* is more abundant than in others, and places where it occurred in the past but are not there anymore, due to over-exploitation. We could also clearly identify social mechanisms and practices focusing in securing the availability of *Agave potatorum*. Local people, in coordination with the authorities of the Tehuacán Biosphere Reserve, about ten years ago started to work in the restoration of *A. potatorum* populations: they sowed seeds in a local greenhouse, when plantlets reach three years of age they were transplanted into the sites of wild agave populations. They started restoring damaged populations of *A. potatorum* and implemented actions to reintroduce plants in populations where the species had disappeared. They also protected large reforested areas using nets to safeguard the plantlets from cattle. Our research team was asked by local authorities to collaborate in monitoring the actions conducted and developing proposals to improve the effectiveness of actions. We designed several studies in order to evaluate the success of transplanting agave plants into the forest, identifying that nearly 90% of plants transplanted to open areas died after one year of being transplanted, whereas most plants under the canopy of shrubs survived. The results of this and other associated studies are published elsewhere but contribute ecological information for improving the population recovery techniques.

The authorities of the Biosphere Reserve established a prohibition to extract *A. potatorum* within the SLA territory, clearly not taking into account the role of *mescal* for the subsistence of people. The total economic benefit from mescal production in SLA is $3,921.57 US dollars per year. Since a total of 60 households (30% of households) are involved in the production process, on average each household received from $64.27 to $450 U.S. dollars per year. This low income is even more drastic since some actors of the mescal production chain receive less income than others. The meager economic benefit and drastic ecological impact easily could lead to the conclusion that it would be cheaper to subsidize the households involved in mescal production than to maintain the destructive production itself. However, the conclusion is in fact not so easy. Mescal is an important part of local customs and culture, even more important than the economic income *per se*. Moreover, because mescal production in any of its steps is only a small part of a diversified peasant economy, the small incomes derived from it and other similar activities supplement the main subsistence activities like agriculture, farming, gathering of wild resources and temporary jobs. A small income represents a significant opportunity to solve specific needs or problems, such as a sudden need of medical care, a family celebration or loss. Mescal is also exchangeable for other subsistence items in local stores and regional markets. Other studies in different regions of Mexico have revealed that similar low economic incomes are fundamental to peasant economy: [36] reported 280 useful plants are collected in SLA from wild populations, but as few as five of these have a commercial or exchange value, *A. potatorum*, *Brahea dulcis* and *B. nitida* appear to be the most important. Rangel-Landa [31] found similar situations with *Brahea* palms in other communities of the Tehuacán Valley.

In order to ensure the *Agave potatorum* availability and keep up the mescal production, about ten years ago some mescal producers started growing and using *Agave marmorata* (*pitzometl*)*,* a local agave that most traditional mescal producers mention would never use. More recently, we have also recorded mescal made from *Agave angustifolia*. This agave has a somehow similar devastating history to that of *Agave tequilana*. The plantation of both was promoted by governmental institutions, cutting down enormous areas of natural vegetation to introduce agave crops for commercial use, replacing local and better adapted agave species with those with poor genetic diversity.

The risk of mescal agave populations disappearing is real, especially in areas surrounding production sites, because the local communitarian institutions did not regulate the extraction of agave plants in the past and because management techniques to prevent their extinction were absent. However, the communitarian authorities started to discuss and regulate how to optimize the use of agave, preventing its disappearing and started a program for its propagation and recovering of populations in collaboration with authorities of the Tehuacán-Cuicatlán Biosphere Reserve and our research group. Our current study allows evaluating the unbalanced relation between availability of *A. potatorum* adult plants and their extraction for mescal production. It is a relation that has been helpful for local people construction of rules and making decision, for protecting and recovering this valuable plant resource.

In this perspective, information derived from our studies allows the following recommendations:

(1) Protection of conserved or low impacted agave populations. Our ecological studies could not identify pristine populations within the territory of SLA but some areas that deserve to be conserved. Furthermore, we recognized that populations of scrublands of *Gochnatia-Dasylirion* and the *Quercus* sp. relicts presented no reproductive individuals and are candidates for restoration and total protection. We suggest that extraction sites like El Machiche and La Cumbre are declared as local conservation areas, at least until the populations recover. Another suggestion is to reintroduce *A. potatorum* in areas once it was present, such as the small hill of La Chirunda located just next to SLA village.

(2) Recovering of impacted populations. People of SLA started sowing seeds in a greenhouse and then transplanting them to the forest. We suggest continuing the efforts of reforestation, transplanting young agaves under nurse plants, based on information generated by [3,4,31].

(3) Plantation of *A. potatorum* in agroforestry systems. Agro forestry systems commonly practiced by local people of the Tehuacán Valley are important reservoirs of biological diversity and have been recognized as key parts of regional strategies of biodiversity conservation [35,36]. Currently, people maintain within these systems several species of *Agave*, among them *A. potatorum*. However, propagation of this species in those systems is difficult since young plants require the protection of nurse plants [31]. It is necessary to develop techniques for simulating effects of nurse plants, like artificial shades, thus favoring the establishment of *A. potatorum* plants in agro forestry systems, far more desirable than the common implementation of agave monocultures.

(4) In order to recover the eroded populations, the monitoring and protection of reproductive individuals of *A. potatorum* is needed, as well as the implementation of a method of annually rotating extraction sites, and sustainable extraction rate of adult plants.

Further ecological studies must be encouraged, for a precise estimation of the availability of agave within SLA territory, in order to explore and propose sustainable extraction rates.

All of these actions should be conducted under the approach of adaptive management [37], which should connect social or local institutions and organizations across levels and scales, such as the Biosphere Reserve, government aid projects and the local actors. Mescal producers are a long distance from working all together, and some seem not to find the advantages of creating an organization, implying equal rights, obligations and benefits to all. Recently, some producers have been working with the Biosphere Reserve introducing young individuals of *Agave marmorata* with young plants from a greenhouse near the village of Zapotitlán, instead of producing agaves in the local greenhouse using local seeds. The introduction of other agave species shows the lack of empathy between the Biosphere Reserve programs and local actors involved in the management of this species. It is a way analogous to promote monoculture models with introduced species, as used in the tequila industry with *A. tequilana* and with *A. angustifolia* in some regions of Oaxaca by industrials of mescal and governmental programs [38]. It also implies changes in the mescal production process, in the spirits organoleptic properties, such as aroma and flavor and yield production. Time will be required by mescal producers people to adapt their preparation techniques to new species with different sugar content and amount of water, time required for fermentation, among other issues. A time of adaptation can represent therefore uncertainty or loss in monetary income.

However, there are some examples, unfortunately still few, of how local *A. potatorum* populations can be recovered with social actions. In Sola de Vega, Oaxaca, Luis Méndez, member of a Rural Production (Union) Society (SPR) *El Solteco*, has worked for more than 10 years in a project to recover and implementing productive crops of this species. *EL Solteco* has already produced mescal with plants from its own greenhouses certificating it products looking forward fare trading. The experience of *El Solteco* may be a model for sustainable use and management of *Agave potatorum* and other agave species and non-timber forest products.

## Conclusions

In SLA *A. potatorum* represents the forest plant resource with higher cultural and economic value, used as food, medicine, fodder, construction material and most important, as main input in mescal production. Our study suggests that current mescal production in SLA is a non-sustainable activity due to its high ecological and social costs and low monetary income.

The remaining populations of *A. potatorum* could support a sustainable use planning, achieved by: protecting conserved agave population such as those under high extraction. It is essential continuing efforts to produce agave plants in the plant nursery of the village. The reforestation and monitoring of these actions as well as of the existing populations must be encouraged. We also consider pertinent to introduce this agave species to agro forestry systems of the area, fallow agriculture areas and known areas where populations disappeared. All these actions require planning of agricultural, forestry and livestock activities through participatory approaches.

## Competing interests

The authors declare that they have no competing interests.

## Authors’ contributions

AD-L main author, involved in the study design, conducted interviews, field work, literature review and general data collection and systematization, wrote the first draft and concluded the final version of this manuscript. AC main coordinator-supervisor of the research project; contributed with original data and the designing of all the researches providing the information for the current analysis; participated in fieldwork, systematization and analysis of data and reviewed several drafts of the manuscript. OT advisor of the studies of vegetation and geographic information system analyses. All authors read and approved the final manuscript.

## Authors’ information

AD postgraduate students at the Centro de Investigaciones en Ecosistemas (CIEco), UNAM. AC full time researcher at CIEco, UNAM. OT full time researcher at UBIPRO, FES Iztacala, UNAM.
